# Clinical indicators of periodontal disease in patients with coronary heart disease: A 10 years longitudinal study

**DOI:** 10.4317/medoral.17848

**Published:** 2012-02-09

**Authors:** Guillermo Machuca, Juan J. Segura-Egea, Gema Jiménez-Beato, Juan R. Lacalle, Pedro Bullón

**Affiliations:** 1 Professor. Department of Special Patients. Faculty of Odontology. University of Seville (Spain); 2 Professor. Department of Endodontics. School of Dentistry. University of Seville (Spain); 3 Assistant Professor. Department of Special Patients. Faculty of Odontology. University of Seville (Spain); 4 Professor. Department of Statistics. Faculty of Medicine. University of Seville (Spain); 5 Professor and Chairman. Department of Periodontics. Faculty of Odontology. University of Seville (Spain)

## Abstract

Objectives: There is evidence about a possible relationship existing between periodontal diseases and coronary heart disease. The aim of the present longitudinal study was to investigate the changes in periodontal evolution after etiological periodontal treatment, comparing a healthy control group with another having coronary heart disease. Study Design: The study included initially 55 patients of which 44 finished it. They were placed into two groups: Healthy Control Group (HCG) n =9, and Coronary Heart Disease Group (CHDG) n=35. The gingival level (GL), probing depth (PD), clinical attachment level (CAL), plaque index (PI) and bleeding on probing (BOP) were measured to compare the periodontal status in both groups. The patients were examined and etiological periodontal treatment was performed and they were then examined at the end of 1 and 10 years. Statistical method: A one way-ANOVA and a MR-ANOVA were established; significance p<0.05. Results: No significant differences between both groups were detected on the first visit (p>0.5). However, at the second visit the CHDG presented a significantly higher PD (p<0.05) and PI (p<0.01). CHDG patients gradually increase PD through time and in comparison to the control group (p<0.041). CHDG patients present a significantly higher CAL loss (p<0.0385) and a significant increase in PI (p<0.0041) at the end of one year, while on the third visit no significant differences were detected in any of these indices. Likewise, a similar fact can be observed on evaluating BOP at the end of ten years causal treatment, a smaller decrease in the cardiac group was observed in regards to the initial values (p<0.001). Conclusion: Patients with coronary heart disease showed a worse evolution of periodontal indices than healthy ones, when referring to probing depth, plaque index and bleeding on probing index.

** Key words:**Attachment loss, coronary heart disease, periodontal disease, risk factors.

## Introduction

Coronary heart diseases (CHD) suppose a major health problem in today’s society in industrialized countries and they are the main cause of mortality in both men and women (1).

Ischemic cardiopathy has a multifactorial origin. The factors which predispose it must fulfil causality criterion: strength of association (relative high risk), consistency of association (demonstrated in several studies), temporal relationship (cause precedes effect), biological acceptability, experimental proof and above all, evidence of studies in humans. From this point of view, the importance that periodontal diseases could have as a risk factor in CHD has been debated (2-4). However, a uniformity of criterion about the impact that periodontal pathology could have on cardiovascular diseases still does not exist. As opposed to the relatively recent cohorts studies, carried out on a great number of patients that associate periodontal disease and death due to cardiovascular diseases (5), or periodontal disease and a high risk of suffering cerebrovascular events (6), there are other equally important and numerous cohorts studies with contrasting results (7-9). The fact must be considered that case-control designed studies, that try to clarify this aspect as well, also present contrasting results. Therefore, greater dental indexes have been found in acute myocardial infarction cases than in the control group (10), while in another similar study on an elderly population, the results did not confirm previous findings (11). Nevertheless, a series of scientific evidence exists which would postulate in favor of said association. Amongst these, the relationship found between periodontitis and atheromatosis is highlighted, in such a way that an increase of the intima of vessels and a higher index of atheromatosis has been described in patients with active periodontitis than in those who do not suffer it (12). In the same way the appearance of periodontal pathogens in cultives of atheromas obtained from endarterectomies by carotid pathology are highlighted (13). Thus Offenbacher et al. have gone so far as to postulate a PAS syndrome (“periodontitis-atherosclerosis syndrome”) which unites both concepts (5). In this sense it would really be important to clarify this concept from the point of view of public health, since to really establish an unmistakable relationship would imply an adequate and precocious foundation of periodontal treatment, reducing the risk of cardiovascular disease in these patients (14).

It is difficult to find long term longitudinal studies in this type of populations, as the difficulty of their planning, and above all the loss of individuals in the study over an extensive period of time can lead to their failure, especially, when bearing in mind these patients’ precarious health. However, when these studies are instituted, they can give quite an enlightening idea about the implications that periodontal disease can have on cardiovascular diseases and vice versa (15). In this way, the present study has been established in which the changes in response to periodontal treatment when a cardiologically healthy control group and another which had undergone coronary diseases are compared.

## Material and Methods

Sample selection

Healthy control group (HCG)

Twelve patients were classified in this group, although only 9 finished the study at 10 years, fulfilling all the requisites. The patients in this group were chosen at random among the ones who applied for a first visit to Department of Periodontology, Dental School, University of Seville for 6 months. All of them had at least two pockets, with probing depths between 3 and 5.5 mm. None of them had previously consumed calcium antagonists, beta-blockers or other medication related to cardiological or immunosuppressor diseases. So as to avoid the presence of non-diagnosed cardiac diseases, all of them were examined at the Cardiology Service at the University Hospital Virgen Macarena of Seville to verify the nonexistence of cardiovascular disease. The study consisted of blood and urine analysis, measurement of arterial pressure, electrocardiogram, auscultation and posteroanterior radiography of the chest. None of these patients developed any cardiological diseases during the ten years.

Coronary heart diseases group (CHDG)

Forty three patients were classified in this group, although only 35 were taken into consideration for the 10 year study, fulfilling all the requisites. Over a 6-month period a group of patients treated at the same Department of Cardiology were asked to take part in the study. All of them had the following common characteristics: (a) they were suffering from some degree of ischemic heart disease; (b) they did not suffer any other relevant systemic pathology; (c) their treatment was based on the use of calcium channel blockers (nifedipine or diltiazem), beta-blockers and coumarin anticoagulants.

In summary, of the 55 patients of both sexes that started the study only 44 finished it, thus the subsequent study refers to these 44 patients. Special precautions were taken so that the patients’ general health was not affected by the study. After informing the patients, each signed an informed consent form. The Ethics and Investigation Committee of the hospital authorized the protocol. Clinical and periodontal examination

Gingival level, clinical attachment level and probing depth

All the participants were examined by the same examiner using a controlled pressure probe (VINE VALLEY RESEARCH MODEL 205, 1037 South Lake Road. Middlesex New York 14507), with which a probing pressure of 177 N/cm could be obtained. The number of teeth present was collected excluding the third molars. Six measures were taken for each tooth, three in vestibular (distal, vestibular, mesial) and another three in palatinum/lingual. Thus three readings were obtained at each site: 1) Gingival level (GL): from the cement um-enamel junction to the gingival margin. 2) Clinical attachment level (CAL): from the cement um-enamel junction line to the most apical area where the probe penetrates. 3) Probing depth (PD): from the free gingival margin to the most apical area of probe penetration.

Plaque index and bleeding on probing

The presence or absence of plaque was collected following the O’Leary et al. (16) plaque index (PI), recorded on the four faces of the tooth. Plaque absence was recorded as zero (0), and presence as one (1).

The bleeding on probing (BOP) index registers absence of (absence=0) or presence of (presence=1) bleeding at the moment of probing, following the index suggested by Van der Velden (17), with the same electronic controlled pressure probe.

All these measurements were recorded at the beginning of the study, at twelve months and at ten years by the same examiner, at double blind, that had previously been calibrated to assure the reproducibility of the study.

On the first visit all the patients were instructed to use the Bass sulcular brushing technique, and interdental brushing using interproximal toothbrushes for oral hygiene. Likewise, root planning and a dental polishing under local anaesthetic were performed, in four sessions, using universal periodontal curets (Columbia 13-14 and 4R-4L). At one year, patients were reviewed again and all the aforementioned measures recorded. The same procedures were carried out at ten years. During the latency period between the last two examinations, patients were recommended to visit their own dentist regularly.

Statistical Analysis

Descriptive statistics of the measurements (Mean and Standard Deviation) were used, calculated for each variable on each visit. The adjustment of comparison between groups was carried out between the basal measurement (1st) and the data from the second and third visit (at one and ten years). When statistically significant differences were established, the Newman-Keuls Student Multiparametric Test was applied. PI and BOP values were expressed referring to the sites of the arch that showed plaques or bleeding in each individual. A one-way analysis of variance test (ANOVA) was used regarding cutting variables. To analyze the changes between visits and the valuation of the different variables (gingival level, pocket, loss of attachment, plaque and bleeding), a repeated measuring analysis of variance (RM-ANOVA) was utilized, considering the normality of variables. To detect whether the consumption of calcium antagonist drugs was interfering in periodontal indices a two-way analysis of variance (ANOVA) was used. However, when any type of significant interaction was detected amongst the different factors (study group or visit), the Wilcoxon multiple comparison test was used, adjusting the signification levels by means of Bonferroni criteria. The established signification level was p<0.05.

Results

 Table 1 shows the periodontal status indicators in the HCG and the CHDG at the 1st visit and at the end of the year (2nd visit). No significant differences between both groups were detected on the first visit (p>0.5). However, at the second visit the CHDG presented a significantly higher PD (p<0.05) and PI (p<0.01). On carrying out a comparison between both groups and visits conjunctly (MR-ANOVA) significant differences are found, so that the coronary cardiopathy group patients gradually increase PD through time and in comparison to the control group (p<0.041). Likewise, a similar fact can be seen on evaluating PI at the end of one year’s causal treatment, a small decrease in the cardiac group being observed in respect to the initial values, as opposed to the healthy control group (p<0.043). On the other hand, significant differences were detected in the evolution between visits, which was not so when evaluating BOP between groups, so a significant reduction (p<0.007) can be seen at the end of a year’s treatment.

**Table 1:**
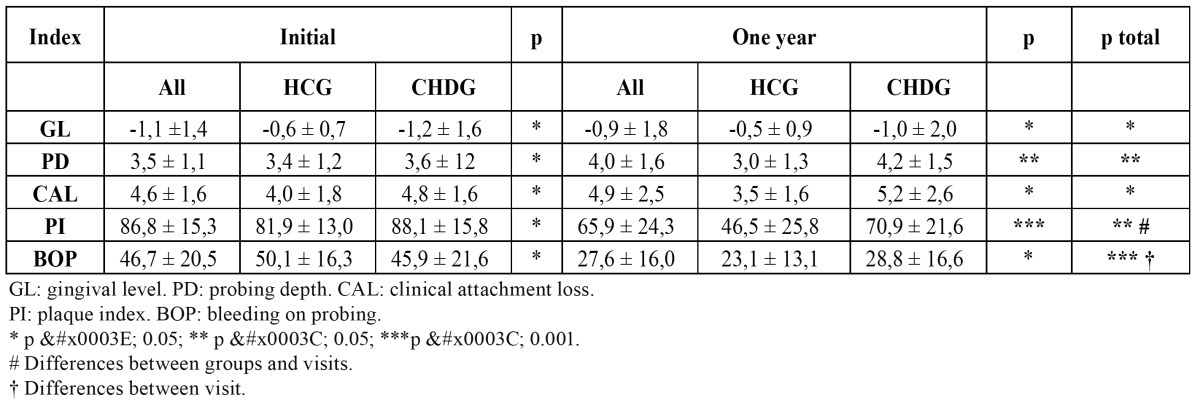
Periodontal status indicators in the healthy control group (HCG) and the coronary heart diseases group (CHDG) referred to the initial and one year visits (n=44).

 Table 2 shows the relationship between the indicators of the periodontal status and the coronary cardiopathy patients and control group on the initial (1st visit), at the end of one year (2nd visit) and at ten years (3rd visit). To evaluate these parameters only patients that were still alive at the end of ten years have been considered. Whereas no significant differences are found when comparing both groups on the control visit, it can be seen how the cardiac patients present a significantly higher CAL loss (p<0.0385) and a significant increase in PI (p<0.0041) at the end of one year, while on the third visit no significant differences were detected in any of these indices. Significant differences were found when comparing both groups and visits conjunctly (MR-ANOVA), in that the patients in the coronary cardiopathy group gradually increased PI through time and in comparison to the control group (p<0.023). Likewise, a similar fact can be observed on evaluating BOP at the end of ten years causal treatment, a smaller decrease in the cardiac group was observed in regards to the initial values (p<0.001).

**Table 2:**
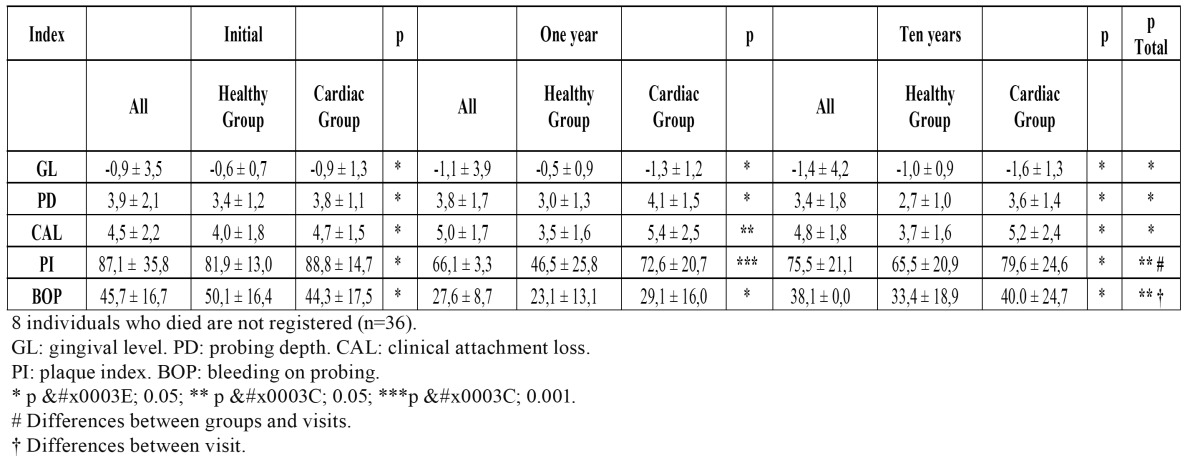
Comparison between healthy control group (HCG) and the coronary heart diseases group (CHDG) referred to first (initial), second (1 year) and third (10 year) visit.

## Discussion

The results of the present study corroborate those established by previous studies (15,18,19), inasmuch that periodontal diseases and coronary diseases have, at least, a tendency to appear together, in such a way that individuals with cardiovascular problems have a greater tendency to have a worse periodontal status and vice versa, as happens in the present study. To what point it is an unequivocal relation remains to be clarified, and for this reason all papers would be justified, as in the present paper, in trying to throw some light on such a transcendental problem of community health.

Over the last few years numerous studies of different designs have been developed (case-control, prospective observational, retrospective observational, metanalysis), which have produced contradictory results when evaluating the association between both diseases (7-18). According to Seymour et al. (19) studies to evaluate the relationship between periodontal and coronary diseases should have a series of characteristics, among which the specific choice of sample to study periodontal problems in cardiological patients, and not the reuse of previous studies for other ends, the use of specific periodontal indices, the fact that the examiners are calibrated and trained and a very careful management of covariables could be underlined. The present study is one of the first longitudinal studies carried out on this problem, which due to its characteristics reflects some of those expressed by Seymour et al. (19). However, the follow-up of such a peculiar group of patients such as these over ten years contemplates a series of difficulties. Amongst these the loss of patients during the period of time (due to deaths, impossibility of locating them, not fulfilling the later visits...) is outstanding. Another problem would be the difficulty of not being able to start with such a large sample as in the cohort studies, as a series of explorations and therapeutic procedures have to be performed on such a large number of patients that the study would be interminable. Yet, despite the results always having to be considered on the basis of particular restrictions of the study, the data they provide can contribute to throw some light on this important problem. This is due to working with a specifically chosen sample, with specific indices for the present periodontal study, taken directly, without being extrapolated from other samples for different purposes, with a unique calibrated and well-trained examiner (to reduce alfa error) and with a certain control over the covariables (medication consumption).

It is interesting to stress some aspects of the study. In the first place, with respect to the choice of sample, it has to be underlined that the patients in the healthy control group were chosen amongst patients who consulted because of suffering some type of periodontal problem, whereas the patients in the cardiological group were selected on the basis of having a cardiac disease, and yet this group has periodontal characteristics comparable to the control group. Thus, while the patients in the control group did not show any cardiac affectation, all the patients in the cardiac group had some kind of periodontal affectation. On the other hand, it is interesting to stress that no significant differences existed in the periodontal situation of individuals with or without cardiopathies at the beginning of the study. The differences began to be seen when evaluating the response to etiological periodontal treatment at the end of one year, when individuals with coronary cardiopathies not only reduced their PD at the end of a year but said index was increased. Likewise, PI and BOP indices were reduced after treatment at a lesser proportion in the cardiac group than in the control group, with a worse response to treatment in cardiopathy patients. This is an important fact, as it would indicate that the cardiopaths increased probing depth in the course of time, with a greater tendency to bleeding on probing, which would indicate the presence of a higher periodontal destructive activity. However, it should always be taken into account that these patients consume platelet antiaggregants that can sometimes alter the results of this index (20,21). It should also be considered that amongst all the evolutions of coronary pathology studied, patients whose illness would lead to death were the ones who showed a significantly worse evolution of probing depth (from 2.96 to 4.58mm, while the control group improved from 3.37 to 2.97mm, the deterioration in the myocardial infarction group was less, from 3.76 to 3.95mm). In fact, when these patients disappeared from the study (Table 2), only PI and BOP showed differences in respect to the healthy control group.

As calcium channel blockers can alter periodontal inflammatory response in different aspects is a demonstrated fact (22,23), the interference that consumption of these drugs of such frequent use in ischemic cardiomyopathy could have in the results of the present study was analyzed. It could be verified that said drugs did not significantly alter the results, so it must be assumed that alterations in the periodontal status shown would not be directly related to the consumption of these drugs.

A high number of studies demonstrate a relationship between the accumulation of bacterial plaque and coronary disease, and above all between the gingival bleeding due to periodontal inflammation and the cardiological status. Thus, Mattila et al. (10), Andriankaja et al. (15), Ying-Ouyang et al. (24), Ramirez et al. (25), Loesche et al. (26) and Morrison et al. (27) find a clear association between these parametres and cardiological deterioration. In the present study it is evident that PI as well as BOP show a sensibly worse evolution in the cardiac group than in the control. Even when the study of patients with worse cardiac evolution disappears, the aforementioned situation is still maintained, so that this would contribute in justifying Wu et al. (6) findings referring to the relationship between the poor periodontal hygiene indicators and the increase in plasma levels of inflammation markers such as C-reactive protein, highly valued in coronary cardiopathy.

In the present study PD mainly increases in the cardiac group, and amongst these in the subgroup that will have a fatal evolution at the end of ten years. These results would be in agreement, in a good proportion, to those of Emingil et al. (28), that find a direct relation between BOP and PD with a worse coronary status (represented by acute myocardial infarction). In the present paper this worse coronary status would be represented by the patients who died as a consequence of coronary cardiopathy, which likewise shows, the worst periodontal status. Nevertheless, it is interesting to stress that when this group of patients, which is going to show a worse evolution, disappears, PD is not going to be a periodontal index with repercussion in the coronary status. It is possible to present the fact that the worst evolution of periodontal status could be related to the most severe cardiopathy patients. In the same way it is possible to highlight the Beck et al. (2) study in which deeper pockets and more acute attachment losses are also associated with ischemic cardiopathy. Geerts et al. (29) ratify this same situation which has also been demonstrated in a woman’s only group by Buhlin et al. (30), in which a worse oral hygiene, an increase in PD and a worsening of cardiological status are associated.

It can be concluded that, in the present study, patients with coronary heart disease have a bad initial periodontal status and presented a worse response to treatment than the control group, especially amongst patients who die as a consequence of their cardiopathy, throughout the study. Nevertheless, although the present study increases indices about the close relationship between a bad periodontal status and a bad response to the etiological phase of periodontal treatment, new studies are needed to be able to definitely clarify the peculiarities of the association between both diseases.
